# Effects of graded talar external rotation on distal tibial articular surface stress and contact pressure: a finite element study

**DOI:** 10.3389/fspor.2026.1810571

**Published:** 2026-06-04

**Authors:** Xinya Lan, JiYuan Mo, Jiaxin Gao, Yaping Li, Guangtao Kang, DanYang He, Tian Li, Zhonghua Lin, Cai Jiang

**Affiliations:** 1 Rehabilitation Medicine Center, Fuzhou University Affiliated Provincial Hospital, Fuzhou, China; 2 College of Rehabilitation Medicine, Fujian University of Traditional Chinese Medicine, Fuzhou, China; 3 Shengli Clinical Medical College of Fujian Medical University, Fuzhou, China

**Keywords:** ankle joint, closed-chain loading, finite element analysis, peak contact pressure, peak surface stress, stress migration, talar external rotation, tibiotalar joint

## Abstract

**Background:**

Abnormal talar biomechanics alter tibiotalar local mechanics and are implicated in several ankle-foot disorders. However, the relationship between graded talar external-rotation moments and local articular changes under controlled loading remains unclear. This study investigated the variation pattern and spatial migration of local mechanical responses on the distal tibial articular surface using a patient-specific three-dimensional finite element ankle model under closed-chain single-leg stance.

**Methods:**

CT data from the right ankle and foot were used to construct a three-dimensional finite element model including cortical bone, cancellous bone, articular cartilage, and major peri-ankle ligaments using Mimics, Geomagic, and SolidWorks. Mild plantarflexion was simulated by a 6° rotation about the line connecting the malleoli. Cartilage contact was defined as low-friction frictional contact, and ligaments were represented by equivalent spring elements. After mesh sensitivity analysis, a 2.0 mm mesh was selected. Fixed support was applied to the plantar surface of the calcaneus, and axial load was distributed between the tibia and fibula at a 5:1 ratio (500 N and 100 N). Graded talar external-rotation moments of 0–5.0 N·m were applied about the tibial long axis. Peak surface stress, peak contact pressure, and relative talar rotation were extracted.

**Results:**

Peak surface stress first decreased and then increased, from 7.56 MPa at 0 N·m to 6.96 MPa at 2.2 N·m, before rising to 8.45 MPa at 5.0 N·m, indicating a relatively low-stress interval within 2.0–2.4 N·m. Peak contact pressure increased continuously from 2.55 MPa to 3.14 MPa, with a slower rise within 0–2.4 N·m and a more pronounced increase beyond 2.6 N·m. Relative talar rotation increased progressively from 0° to 1.61°. Both peak surface stress and peak contact pressure migrated overall from the anterolateral to the anteromedial tibial plafond.

**Conclusion:**

Under closed-chain single-leg stance loading, talar external rotation redistributes the local mechanical environment of the distal tibial articular surface. Peak surface stress shows a relatively low-stress interval, whereas peak contact pressure increases overall. These findings suggest that talar external rotation alters both local stress levels and the location of the primary tibiotalar contact zone, and should therefore be interpreted using multiple mechanical indicators.

## 1 Introduction

The talus is a key load-bearing bone within the ankle mortise. Once its alignment or biomechanical state becomes abnormal, the contact area and peak contact stress of the tibiotalar joint may change markedly, thereby reshaping the local mechanical environment and becoming closely related to the onset and progression of various ankle and foot disorders (1). Disorders such as osteochondral lesion of the talus (OLT), chronic ankle instability (CAI), and adult acquired flexible flatfoot deformity (AAFD) are often accompanied, at different stages of their clinical course, by talar malalignment or biomechanical abnormalities, which in turn induce redistribution of tibiotalar contact stress and cumulative injury to the articular cartilage and subchondral bone (2–5). Clinically, such conditions are commonly associated with pain, joint dysfunction, and degenerative cartilage changes, and in severe cases may further compromise long-term joint stability and function, imposing a substantial burden on patients’ daily activities and mobility (6–8).

To address these mechanical problems, clinical practice often uses medial foot elevation to induce an appropriate degree of inversion, thereby reducing abnormal ankle contact loading during walking or weight-bearing and partially correcting structural and functional abnormalities (9). Previous related studies have mainly focused on changes in lower-limb alignment, plantar pressure distribution, gait parameters, and overall joint contact characteristics, whereas quantitative studies specifically targeting local peak stress and its spatial migration on the distal tibial articular surface remain relatively limited (10). Although some studies have used CT-based finite element analysis to investigate the influence of multidirectional talar displacement on tibiotalar contact mechanics, systematic analyses of the dose-response relationship between graded external-rotation moments and local articular mechanical changes under controlled loading conditions are still scarce, and direct investigation of a potential “minimum stress window” remains insufficient.

Because local articular stress is difficult to measure directly *in vivo* and traditional experimental methods have difficulty achieving continuous comparisons across multiple loading levels under unified conditions, finite element models provide an important tool for revealing internal ankle joint stress distributions (11, 12). Accordingly, the present study used a three-dimensional finite element model under a single-leg stance loading condition and applied graded external-rotation moments to the talus to simulate a specific biomechanical perturbation. We focused on the variation and spatial migration characteristics of peak surface stress and peak contact pressure on the distal tibial articular surface and compared the differential responses of these two indicators to external-rotation perturbation, with the aim of providing parameter-level biomechanical evidence for ankle and foot disorders that share abnormal talar biomechanics as a common pathological feature.

## 2 Materials and methods

### 2.1 Materials

One healthy 25-year-old male volunteer was included in this study (height: 173 cm; body mass: 60 kg), with no prior history of ankle trauma. Imaging examinations excluded tumors, deformities, fractures, and other organic lesions of the foot and ankle. Written informed consent was obtained from the participant, and the study protocol was approved by the institutional ethics committee.

The participant underwent CT scanning of the right ankle and foot at Fujian Provincial Hospital affiliated with Fuzhou University. Scanning was performed using a NeuViz Epoch CT scanner, with a voxel size of 0.834 mm^3^ × 0.834 mm^3^ × 1.0 mm^3^. The resulting images were exported in DICOM format for subsequent three-dimensional reconstruction and finite element modeling. Mimics Medical 21.0 was used for medical image processing and three-dimensional reconstruction.

### 2.2 Data acquisition

CT scanning of the participant's right ankle-foot complex was performed by a radiologic technologist with more than 5 years of experience in the radiology department of a provincial tertiary hospital. Before scanning, a non-weight-bearing support device was placed under the plantar surface to maintain the ankle and foot in a neutral position. The scan range extended from the distal one-third of the tibia to the entire foot so as to fully capture the bony structures of the ankle and foot. After acquisition, the image data were exported and stored in DICOM format for subsequent image segmentation, geometric reconstruction, and finite element model establishment.

### 2.3 Construction of the finite element model of the ankle joint

**Figure 1:**
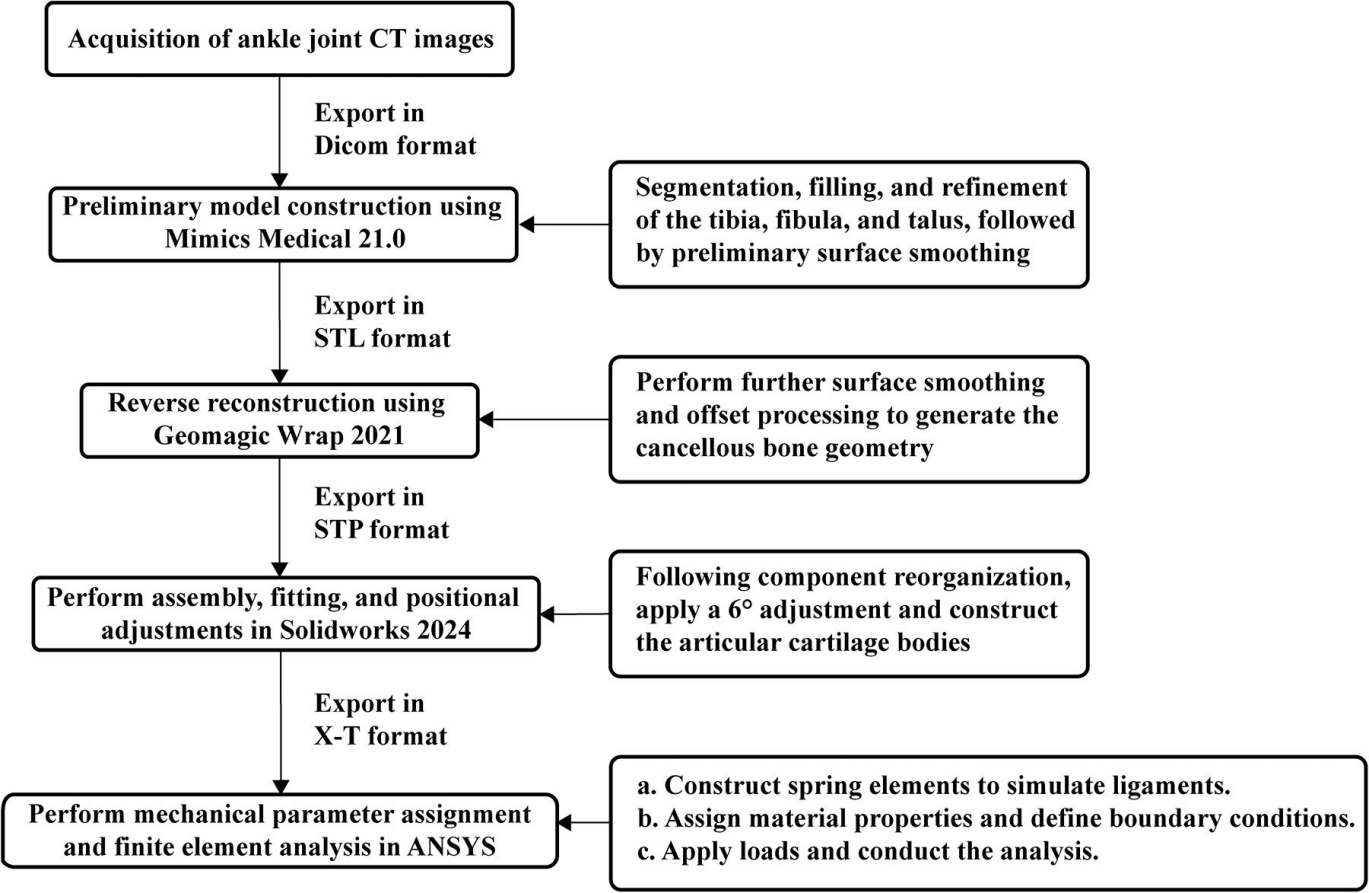
Workflow for the establishment and analysis of the ankle finite element model.

The three-dimensional finite element modeling and analysis workflow of this study consisted of four stages (Figure 1): acquisition of ankle CT images and export of DICOM data; segmentation of bone and articular cartilage, geometric reconstruction, and model refinement; finite element assembly together with assignment of ligament, cartilage, and other structural properties and mesh generation; and, finally, definition of boundary conditions and loads followed by numerical contact stress analysis. The methods used in each stage are described in detail below.

**Figure 2:**
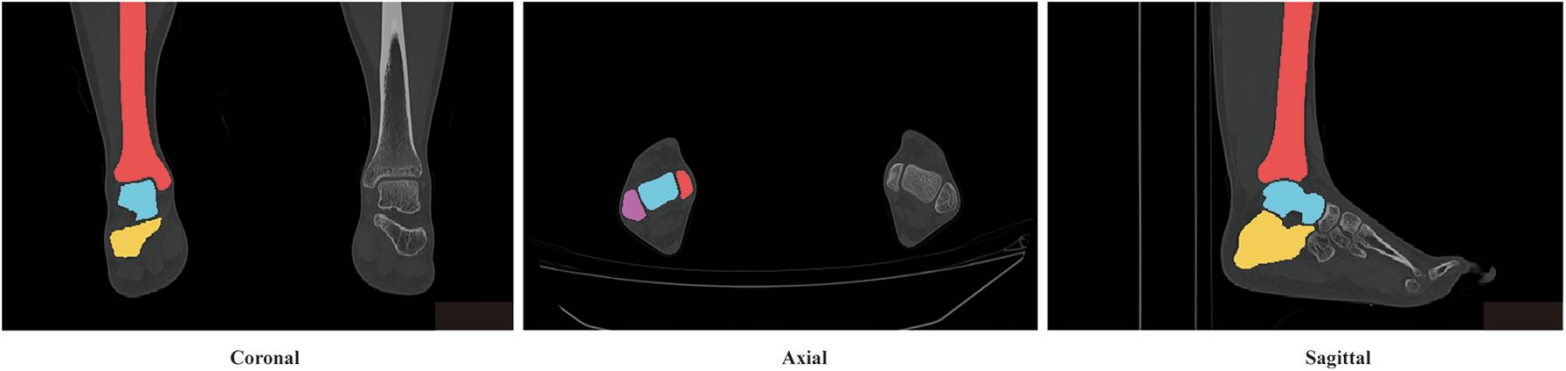
Mimics segmentation schematic. Coronal, axial, and sagittal views of the segmented bony structures.

1. **Initial model generation and export:** After importing the DICOM data into Mimics Medical 21.0, initial segmentation of the tibia, fibula, talus, and calcaneus was performed based on grayscale thresholding, followed by layer-by-layer manual correction to complete contour extraction. During segmentation, tracing was mainly performed along the cortical outlines and bony boundaries identifiable on CT images so as to preserve the original anatomical morphology as much as possible. After segmentation, local holes, discontinuous boundaries, and surface burrs caused by image resolution and thresholding errors were repaired and smoothed as needed, including filling of small holes, removal of isolated fragments, and local surface optimization, without altering the major bony contours or the overall morphology of the articular surfaces. Mimics segmentation schematics are shown in Figure 2, including the segmentation results in the coronal, axial, and sagittal views.
2. **Reverse reconstruction and cancellous bone modeling:** Geomagic Wrap 2021 was used for reverse reconstruction of the ankle STL data. On this basis, a 1-mm inward offset was applied to the tibia, fibula, talus, and calcaneus to generate the cancellous bone geometries (13). Similar strategies for generating cortical and cancellous bone based on contour offset have been adopted in previous foot and ankle finite element studies, providing methodological support for the simplification used here (14).
3. **Model assembly and construction of cortical bone and articular cartilage:** The geometries were imported into SolidWorks 2024 for assembly and optimization, and cortical bone shells with a thickness of 1 mm were constructed for each bony segment. To simulate the mild plantarflexed posture induced by heel elevation and to maintain a reasonable alignment of the talus within the ankle mortise formed by the tibia-fibula complex, the ankle was adjusted to mild plantarflexion during assembly by rotating 6° about the line connecting the anatomical landmark points of the medial and lateral malleoli. Previous *in vivo* biplanar fluoroscopy studies have shown that during plantarflexion the talus may translate anteriorly, allowing its narrower posterior portion to engage further into the ankle mortise (15). Therefore, the talus was translated relative to the tibia according to their positional relationship to ensure appropriate placement within the mortise and overall geometric fidelity of the ankle model. Subsequently, articular cartilage solids were generated by extending the articular surfaces of the relevant bones outward. Because the true cartilage boundary cannot be reliably identified on CT, separate double-layer cartilage reconstructions for apposing joint surfaces were not performed. Instead, referring to previous foot finite element studies, simplified continuous articular cartilage bodies were generated based on the geometric region between adjacent bony articular surfaces, and these were used to analyze relative mechanical responses of the joint surfaces under different external-rotation moments (16). In line with previous foot and ankle finite element studies, articular cartilage was simplified as a homogeneous, isotropic, linear elastic material for comparative biomechanical analysis under controlled loading conditions (16).
4. **Finite element setup and assignment of material properties:** The solid models were imported into ANSYS 21 for model setup and computation. Bones and articular cartilage were meshed using quadratic tetrahedral solid elements to accommodate the complex irregular geometry of the foot and ankle. Material properties were assigned according to the anatomical and biomechanical characteristics of each tissue, based on prior studies and recent developments in the field (17–19), as summarized in Table 1. Bone was modeled as an isotropic, linear elastic material. Because articular cartilage is characterized by a very low friction coefficient (<0.0025), cartilage-related contact behavior across the opposing articular surfaces was defined as low-friction frictional contact with a coefficient of friction of 0.0025 (20). Except for the articular contact interfaces and the ligament spring connections described above, all remaining interfaces in the model were defined as bonded contacts.

**Table 1:** Material properties of the components of the finite element model.

Material	Young's modulus (MPa)	Poisson's ratio
Cortical bone (18)	17,000	0.3
Cancellous bone (18)	700	0.2
Articular cartilage (17)	10	0.4

**Figure 3:**
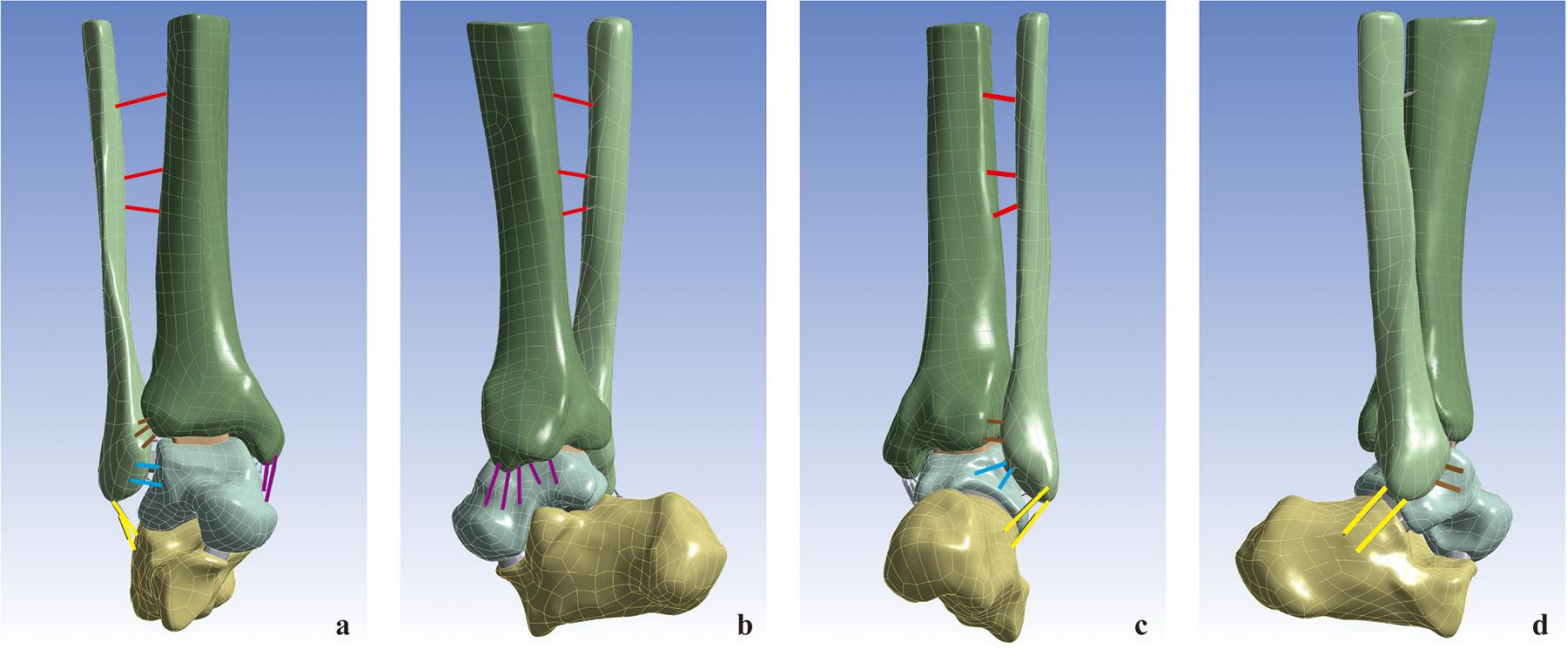
Locations of ligament constructions in the finite element model **(a)** anterior view; **(b)** medial view; **(c)** posterior view; **(d)** lateral view Red: interosseous tibiofibular ligament; blue: talofibular ligaments (anterior and posterior); brown: inferior tibiofibular ligaments (anterior and posterior); purple: tibiotalar ligaments (anterior and posterior); yellow: calcaneofibular ligament.

Because this study mainly focused on the relative changes of articular mechanical responses under different external-rotation moments, the peri-ankle ligaments were simplified as equivalent spring connections established according to anatomical origin and insertion sites, with stiffness parameters selected from the literature to represent their principal stabilizing role; more complex features such as nonlinear constitutive behavior, prestress, and viscoelasticity were not explicitly reconstructed (21, 22). The modeled peri-ankle ligaments included the anterior tibiotalar ligament, posterior tibiotalar ligament, anterior talofibular ligament, posterior talofibular ligament, anterior inferior tibiofibular ligament, posterior inferior tibiofibular ligament, and interosseous tibiofibular ligament. Based on Netter's atlas (23), the anatomical origin and insertion of each ligament were identified, and corresponding locations were selected on the model to establish spring ligaments (Figure 3). Ligament connectivity was thus simulated, and the stiffness of each spring set according to previously reported values (Table 2), Ligament stiffnesses were primarily adopted from previously published ankle finite element parameter sets. For ligaments represented by multiple spring elements, the assigned spring stiffnesses were distributed such that their sum equaled the total ligament stiffness. For the ATFL, the two spring elements were assigned unequal stiffnesses (108.6 and 33.2 N/mm) to reflect the superior and inferior fascicles reported in the reference model (24–27).

**Figure 4:**
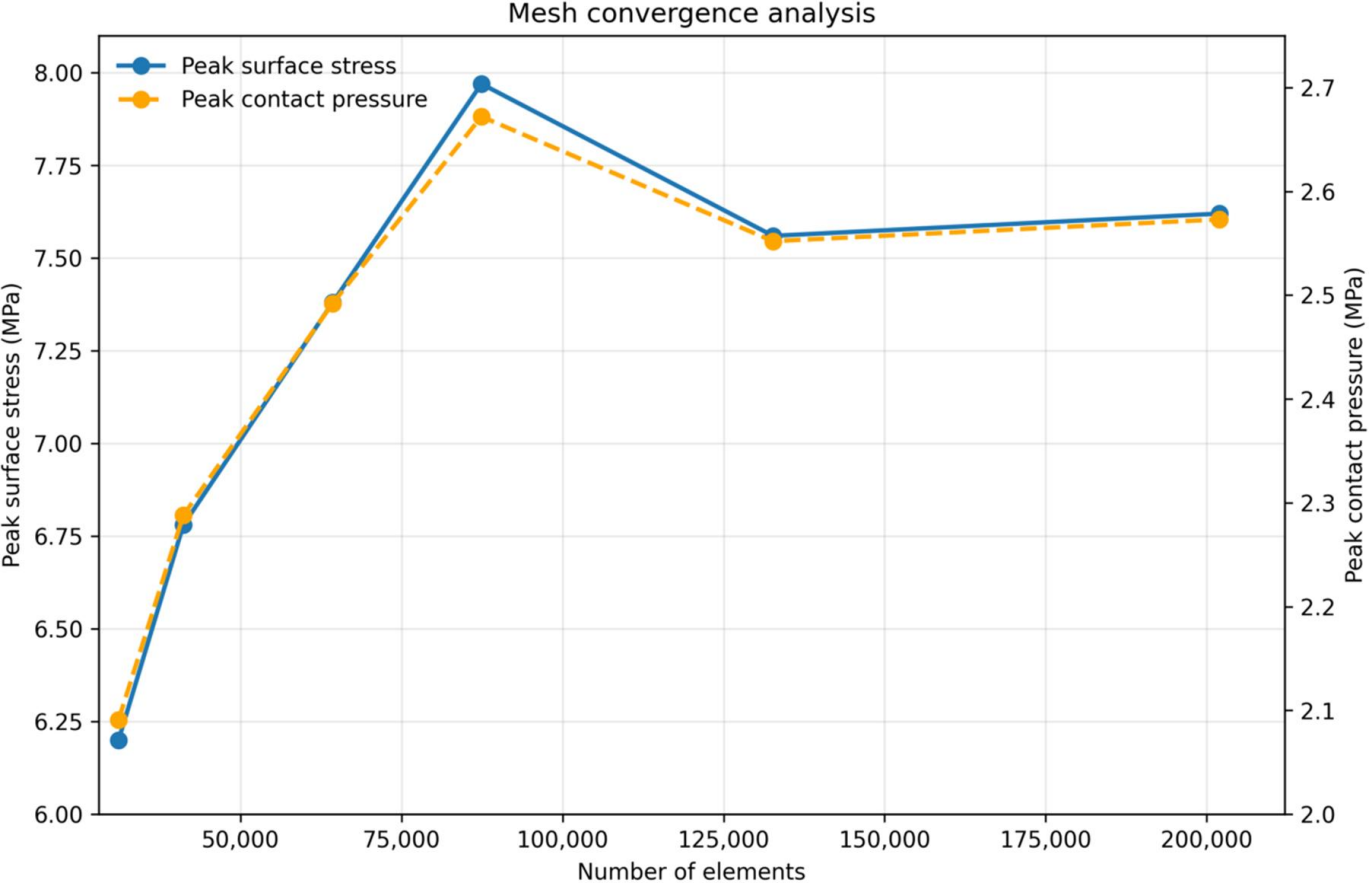
Mesh convergence analysis of peak surface stress and peak contact pressure on the distal tibial articular surface.

**Figure 5:**
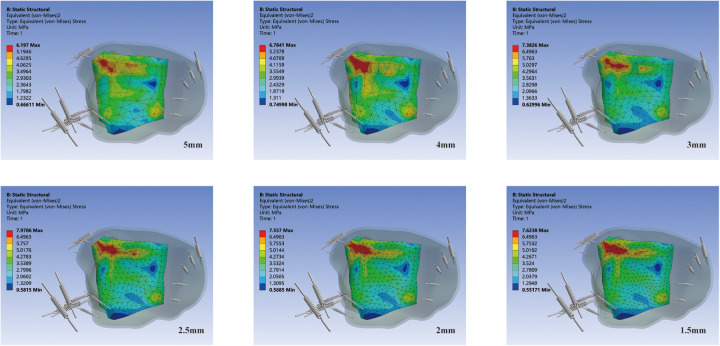
Articular surface stress distributions under different mesh densities.

**Table 2:** Stiffness parameters of the major ankle ligaments.

Ligament	No. of spring elements	Total stiffness (N/mm)	Stiffness allocation (N/mm)	Reference
Anterior tibiotalar ligament	3	122.6	40.87 × 3	Ji et al. (27); Wu et al. (26)
Posterior tibiotalar ligament	2	60	30.00 × 2	Ji et al. (27); Wu et al. (26)
Anterior talofibular ligament (ATFL)	2	141.8	108.6 + 33.2	Ji et al. (27); Wu et al. (26)
Posterior talofibular ligament (PTFL)	1	82	82.00 × 1	Ji et al. (27); Wu et al. (26)
Anterior inferior tibiofibular ligament (AITFL)	3	78	26.00 × 3	Ji et al. (27);
Posterior inferior tibiofibular ligament (PITFL)	2	101	50.50 × 2	Ji et al. (27); Beumer et al. (24)
Calcaneofibular ligament (CFL)	2	80	40 × 2	Ji et al. (27); Beumer et al. (24)
Interosseous tibiofibular ligament	3	234	78.00 × 3	Wu et al. (26); Hoefnagels et al. (25)

### 2.4 Verification and validation

#### 2.4.1 Mesh sensitivity analysis

The intact ankle model was discretized into six mesh densities with a respective average element side length of 5.0 mm, 4.0 mm, 3.0 mm, 2.5 mm, 2.0 mm, and 1.5 mm to perform a mesh convergence study. The same boundary conditions and loading were applied to all mesh density models, including fixed support at the plantar surface of the calcaneus (28), remote displacement at the distal tibia and fibula restricting translation and rotation about the *X* and *Y* axes, and a 600 N axial load applied to the distal tibia-fibula complex. Peak surface stress and peak contact pressure on the distal tibial articular surface were compared across mesh densities. The numerical results are summarized in Table 3, the corresponding mesh convergence curve is shown in Figure 4, and representative articular surface stress distributions under different mesh densities are shown in Figure 5. With progressive mesh refinement, peak surface stress increased from 6.20 MPa with the 5.0 mm mesh to 7.97 MPa with the 2.5 mm mesh, and then tended to stabilize in the fine-mesh range. Further comparison showed that peak surface stress was 7.56 MPa with the 2.0 mm mesh and 7.62 MPa with the 1.5 mm mesh, a relative difference of only 0.88%. A similar trend was shown for peak contact pressure, which was 2.091 MPa with the 5.0 mm mesh and increased to 2.552 MPa with the 2.0 mm mesh, corresponding to a relative difference of only 0.82%. Considering that further refinement from 2.0 mm to 1.5 mm increased the number of nodes from 251,816 to 381,074 and the number of elements from 132,675 to 201,993, while the primary endpoint changed only slightly, a 2.0 mm mesh was selected for subsequent analysis in order to balance computational accuracy and efficiency (29).

**Table 3:** Numbers of model nodes elements and peak stress results under different mesh densities.

Grid level	Nodes	Elements	Peak surface stress (MPa)	Peak contact pressure (MPa)
5.0 mm	60,333	31,068	6.20	2.091
4.0 mm	79,452	41,117	6.78	2.288
3.0 mm	123,214	64,300	7.38	2.492
2.5 mm	166,679	87,398	7.97	2.672
2.0 mm	251,816	132,675	7.56	2.552
1.5 mm	381,074	201,993	7.62	2.573

#### 2.4.2 Baseline model plausibility under the 0 N·m condition

**Figure 6:**
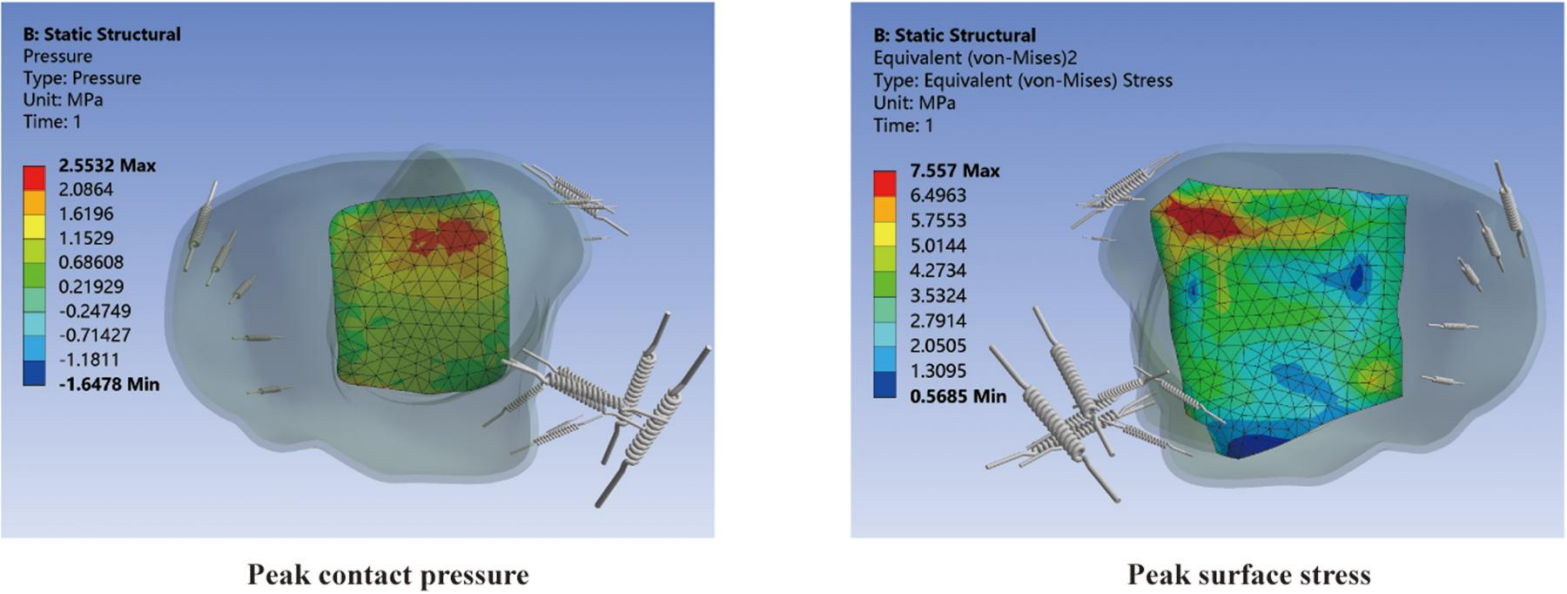
Stress contour map of the distal tibial articular surface.

Under the 0 N·m condition, peak surface stress on the distal tibial articular surface was 7.56 MPa, peak contact pressure was 2.553 MPa, and the contact area of the distal tibial articular surface was 453.12 mm^2^. Reported peak contact stress/pressure values in normal or intact ankles vary across studies, mainly due to differences in loading methods, ankle posture, and subjects (Table 4). The peak pressure obtained here lies in the low-to-moderate part of the reported range, being closer to values reported under static compression or relatively low load conditions and lower than those reported under high-load gait or dorsiflexed conditions. Considering that the present model used a static closed-chain compression condition together with simplified soft-tissue and boundary settings, the obtained value is overall within an acceptable and reasonable range (Table 4 and Figure 6) (29–32).

**Table 4:** Reported ankle joint stress values in previous studies.

Study	Peak contact pressure (MPa)
Present study	2.55
Anderson et al. (29)	3.69
Vrahas et al. (32)	1.9–12.4
Li et al. (31)	7.4–12.9

## 3 Boundary conditions and loading

A structured meshing strategy was adopted, with the element size for cortical bone, cancellous bone, and articular cartilage set to 2 mm. Fixed support was applied to the full plantar bony surface of the calcaneus represented in the model, as a simplified surrogate of ground support under the present static closed-chain loading condition (28). Local coordinate systems were established at the proximal tibia and fibula, with the *Z*-axis aligned along the proximal-to-distal shaft direction and the *X* and *Y*-axes lying in the transverse plane perpendicular to *Z* and orthogonal to each other. Because the present loading and result interpretation were mainly centered on the axial direction, the *X* and *Y*-axes served primarily as auxiliary directions for defining remote displacement and rotational constraints. Given that the present model did not explicitly include the knee joint, the rotational influence of the proximal tibia-fibula complex within the overall lower-limb mechanical chain was simplified through remote displacement constraints. Remote displacement was then applied to the ankle-foot model. In the loading setup, based on biomechanical studies reporting that the fibula carries approximately one-sixth of the static axial load, a tibia-to-fibula load-sharing ratio of 5:1 was prescribed, with 500 N applied to the tibia and 100 N to the fibula, respectively (33, 34). It should be noted that the load share carried by the fibula may vary with ankle position and experimental conditions; therefore, this ratio was adopted here as a unified simplified boundary condition.

**Figure 7:**
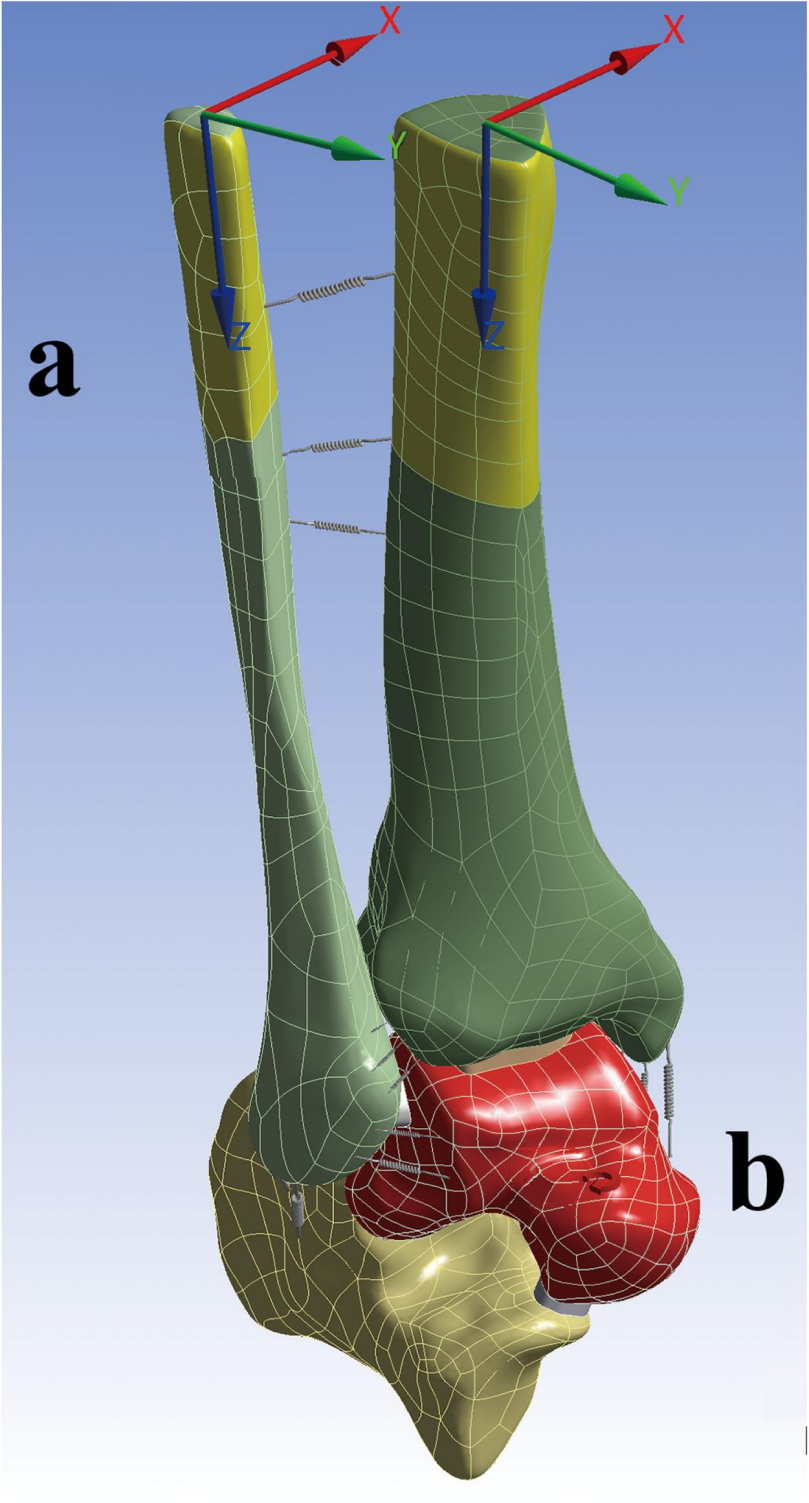
Schematic of the model coordinate system and boundary conditions **(a)** region where remote displacement was applied; **(b)** region where the moment was applied.

Previous studies have suggested that, under a closed-chain standing condition with restricted plantar contact, changes in hindfoot coronal-plane posture may be coupled with axial rotation of the talus within the ankle mortise; that is, certain kinematic components of foot “inversion” may be accompanied by talar external rotation (35). Based on this closed-chain coupling concept, the present study used graded talar external-rotation moments as surrogate intervention variables. Specifically, while keeping the single-leg stance boundary conditions and load-sharing scheme unchanged, an external-rotation moment gradient of 0–5 N·m was applied to the talus about the tibial long axis in order to characterize the response curves and spatial migration patterns of mechanical indicators on the distal tibial articular surface (Figure 7). Here, 4 N·m was selected as the commonly used upper-limit benchmark for external-rotation stability testing reported in the literature, and an additional 1 N·m interval (4–5 N·m) was included for safety-boundary exploration (36).

In the present study, a closed-chain loading condition refers to a constrained stance configuration in which the foot remains in contact with the supporting surface and load is transmitted proximally through the ankle-foot complex rather than being applied in a free distal-end condition. Peak surface stress was defined as the peak von Mises stress on the distal tibial articular surface, whereas peak contact pressure was defined as the peak normal contact stress at the tibiotalar contact interface.

## 4 Results

### 4.1 Trends in peak surface stress and peak contact pressure at the distal tibial

With the single-leg closed-chain boundary conditions and load-sharing scheme held constant, increasing the talar external-rotation moment from 0 N·m to 5.0 N·m produced different response patterns for peak surface stress and peak contact pressure on the distal tibial articular surface (Table 5).

**Table 5:** Stress-related metrics under different external-rotation moments.

Moment (N·m)	Peak surface stress (MPa)	Peak contact pressure (MPa)	Delta relative rotation angle (°)
0	7.56	2.553	0.00
0.2	7.49	2.554	0.06
0.4	7.42	2.555	0.13
0.6	7.35	2.556	0.19
0.8	7.28	2.557	0.26
1.0	7.22	2.559	0.32
1.2	7.17	2.560	0.39
1.4	7.12	2.561	0.45
1.6	7.08	2.562	0.52
1.8	7.04	2.564	0.58
2.0	7.00	2.565	0.64
2.2	6.96	2.566	0.71
2.4	6.98	2.567	0.77
2.6	7.01	2.597	0.84
2.7	7.05	2.619	0.87
2.8	7.10	2.642	0.90
3.0	7.21	2.687	0.97
3.5	7.48	2.799	1.13
4.0	7.76	2.912	1.29
4.5	8.05	3.024	1.45
5.0	8.45	3.137	1.61

**Figure 8:**
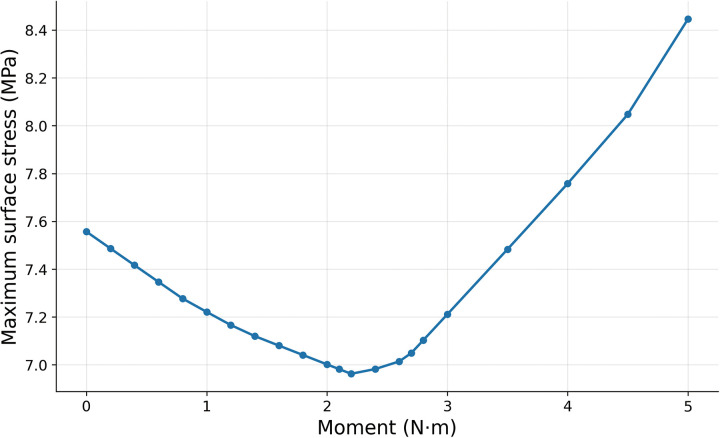
Peak surface stress curve.

Regarding peak Surface Stress, the overall pattern showed an initial decrease followed by an increase. Within the range of 0–2.2 N·m, peak surface stress decreased progressively from 7.56 MPa to 6.96 MPa, representing a reduction of approximately 7.87%, with the minimum reached at 2.2 N·m. Thereafter, as the applied moment continued to increase, peak surface stress rose again and reached 8.45 MPa at 5.0 N·m, which was approximately 11.77% higher than the baseline (0 N·m). Numerically, peak surface stress remained at a relatively low level within 2.0–2.4 N·m, suggesting that this interval may correspond to a relatively stable low-stress range (Figure 8).

**Figure 9:**
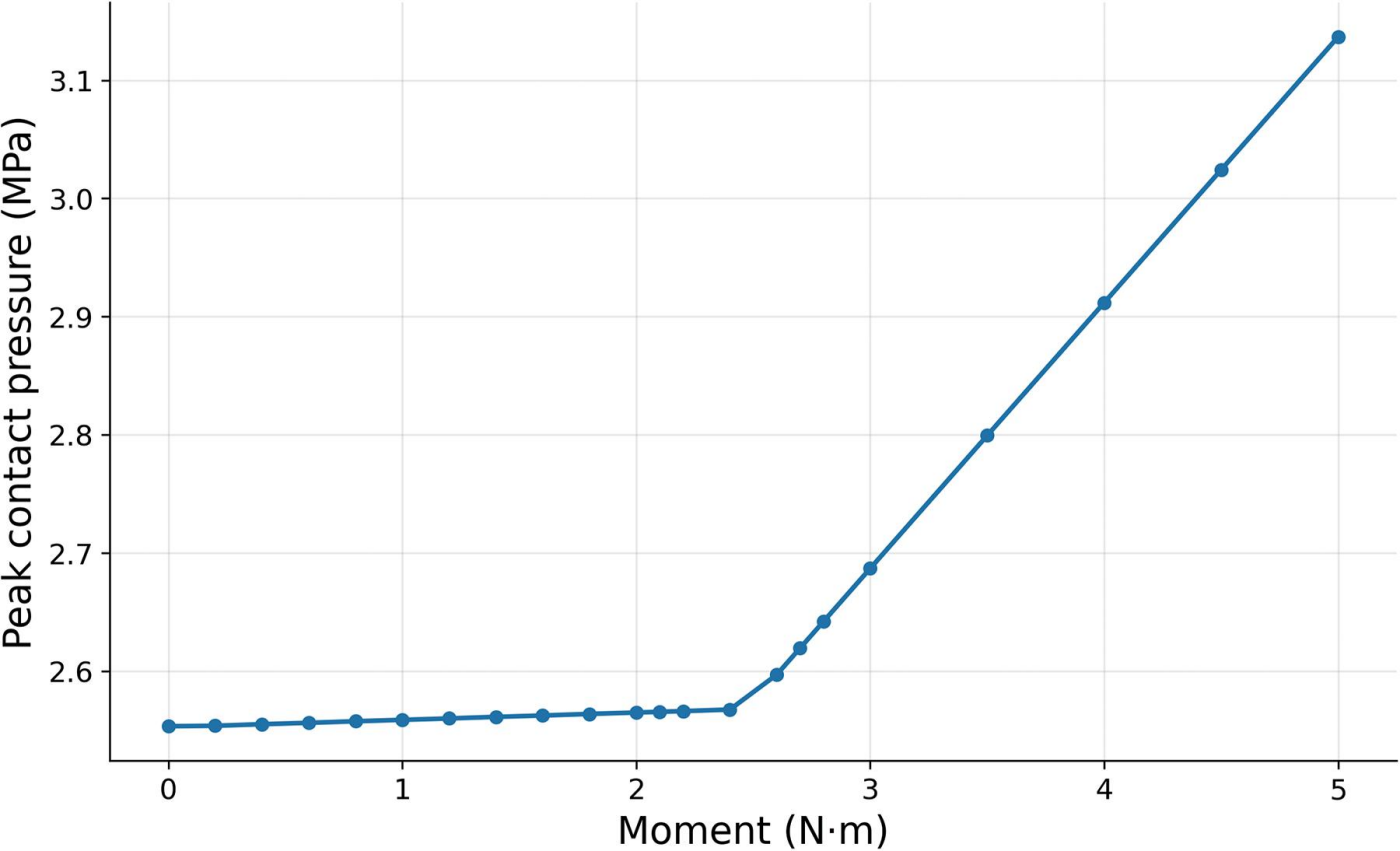
Peak contact pressure curve.

In contrast, peak contact pressure showed a continuously increasing trend with increasing external-rotation moment. It rose from 2.553 MPa at 0 N·m to 3.137 MPa at 5.0 N·m, corresponding to an overall increase of approximately 22.86%. During the low-moment phase (0–2.4 N·m), the increase in peak contact pressure was relatively gradual, whereas beyond 2.6 N·m the upward trend became more pronounced, indicating progressive intensification of local contact-load concentration under higher external-rotation moments (Figure 9).

**Figure 10:**
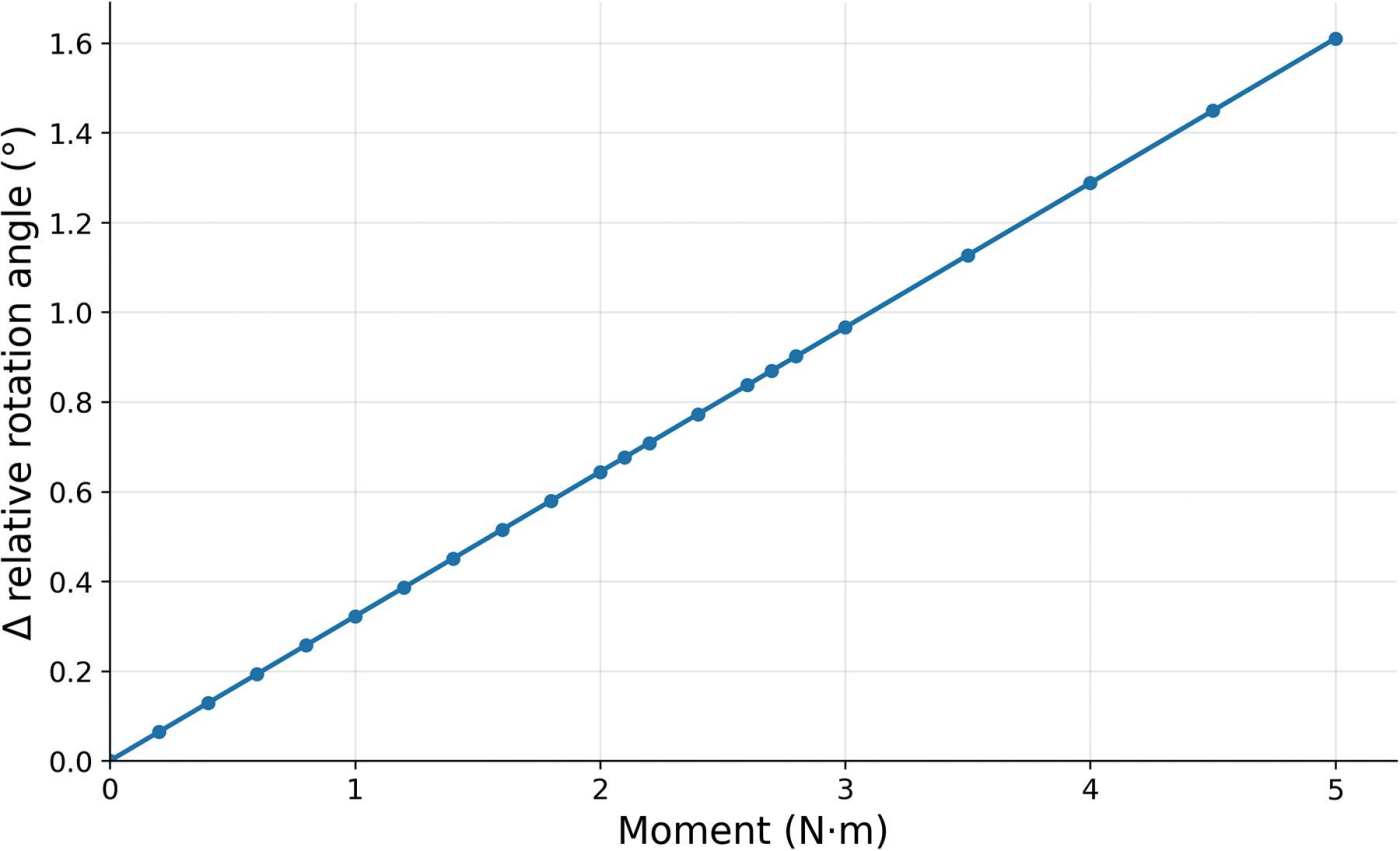
Change in relative rotation angle.

In addition, using the 0 N·m condition as baseline, the change in relative rotation angle of the talus with respect to the tibia about the *Z* axis increased progressively from 0° to 1.6100° at 5.0 N·m, showing a continuous and monotonic pattern with increasing moment. This finding indicates that, under the present closed-chain compressive constraint, graded external-rotation moments produced stable and measurable axial rotational responses, thereby providing a kinematic basis for the subsequent changes in articular stress and contact pressure (Figure 10). Calek et al. reported slight increases in external-rotation–related angular measurements with increasing applied torque in healthy ankles (median ER: 12.2° at 0 Nm and 12.9° at 5 Nm), whereas the present model showed a 1.61° increase in relative talar rotation at 5 N·m, indicating a mechanically plausible rotational response, although the angular definitions were not identical (37).

### 4.2 Spatial migration characteristics of peak surface stress distribution at the distal tibial articular surface

**Figure 11:**
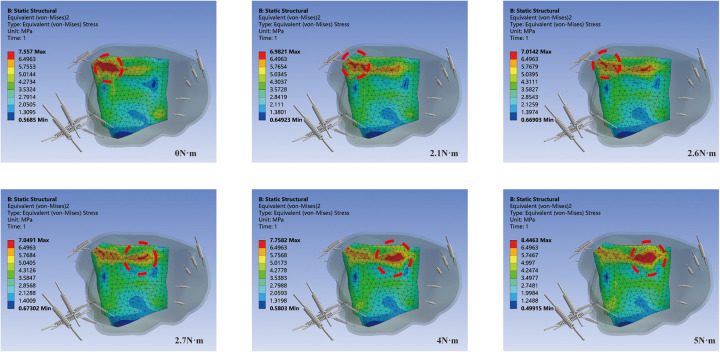
Peak surface stress (red circles indicate peak-stress regions).

According to the peak surface stress contour maps of the distal tibial articular surface (Figure 11), increasing talar external-rotation moments caused the high-stress region to migrate overall from the anterolateral to the anteromedial region. Under the 0 N·m condition, peak stress was mainly concentrated in the anterolateral region, indicating that local surface stress initially concentrated in the lateral anterior aspect at baseline. As the moment increased to 2.1 N·m, the high-stress region extended medially along the anterior margin, and the high-value band became more continuous than at baseline, suggesting redistribution of anterior articular surface stress during the low-to-moderate moment phase. At 2.6 N·m, although the high-stress region still extended along the anterior edge as a whole, the stress hotspot remained mainly on the lateral side; when the moment further increased to 2.7 N·m, the hotspot corresponding to the maximum stress shifted to the medial side, indicating that this interval represented the key transition stage during which the dominant region of maximum stress switched from lateral to medial. As the moment continued to increase to 4.0 N·m and 5.0 N·m, the high-stress region in the anteromedial area became further intensified and more localized, indicating that under higher moments the articular surface stress not only increased overall but also became more concentrated locally.

Overall, the spatial migration pattern of peak surface stress on the distal tibial articular surface can be summarized as follows: the high-stress region progressively migrated from the anterolateral to the anteromedial area, with 2.6–2.7 N·m representing the key interval during which the peak hotspot shifted from lateral to medial. Combined with the numerical results, peak surface stress remained at a relatively low level within 2.0–2.4 N·m, suggesting that this interval may be regarded as a relative minimum-stress window for the articular surface.

### 4.3 Spatial migration characteristics of peak contact pressure distribution at the distal tibial articular surface

**Figure 12:**
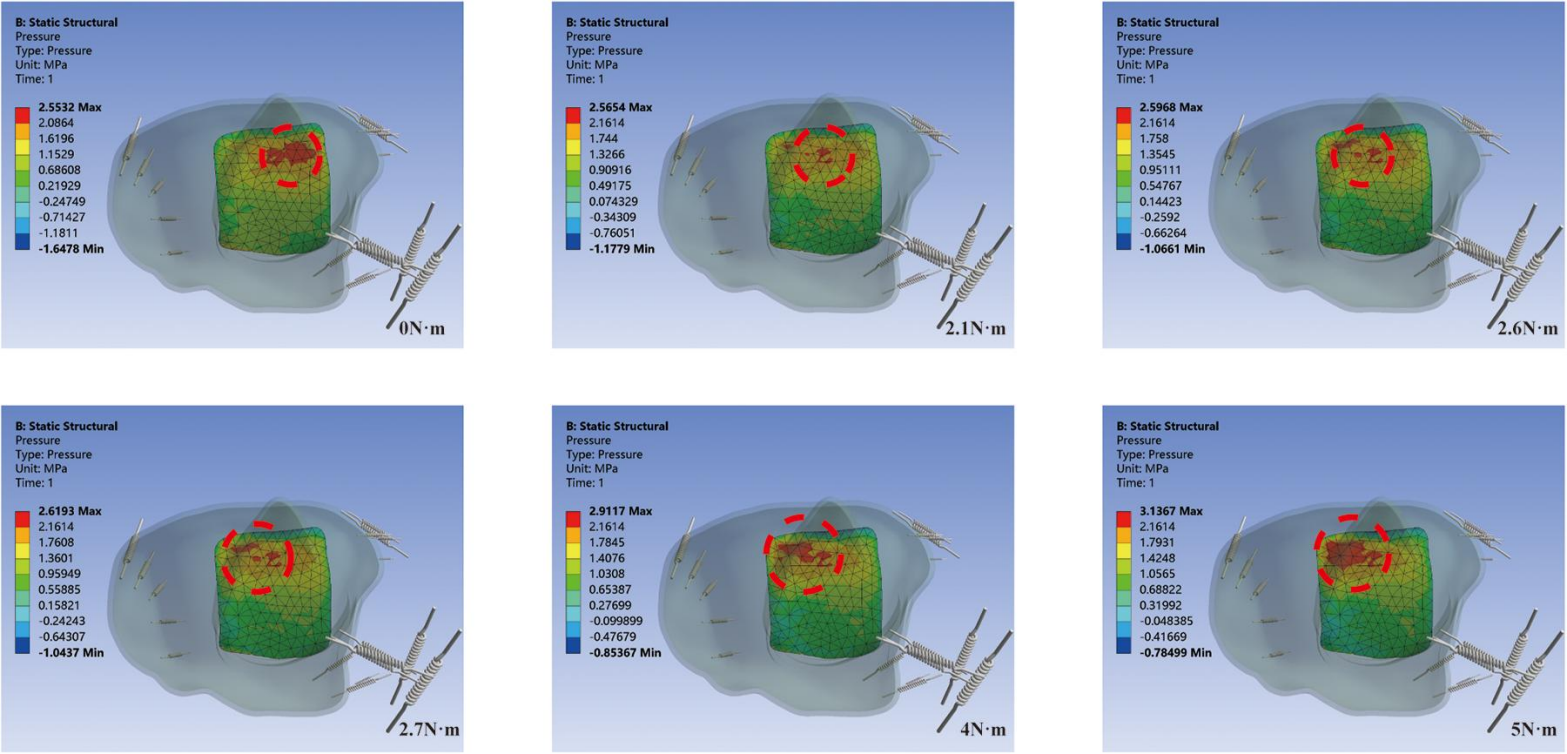
Peak contact pressure (red circles indicate peak-stress regions).

Compared with peak surface stress, the spatial migration of peak contact pressure was more clearly defined. The pressure contour maps showed that, with increasing external-rotation moment, the region of peak contact pressure gradually migrated from lateral to medial while local high-pressure concentration progressively increased (Figure 12). Under the 0 N·m condition, peak contact pressure was mainly located in the anterolateral region, indicating that the contact load was initially concentrated in the lateral anterior region. As the moment increased to 2.1 N·m, the high-pressure region remained along the anterior articular margin, but its center shifted slightly medially relative to baseline. At 2.6 N·m and 2.7 N·m, the hotspot of peak contact pressure migrated further medially, suggesting that with continued talar external rotation, the principal load-bearing contact region gradually shifted from the anterolateral toward a more anteromedial location. When the moment increased to 4.0 N·m and 5.0 N·m, this migration became even more evident. The high-pressure region not only concentrated further medially, but the extent and intensity of the local red high-value area also increased substantially, indicating that under higher moments the articular contact load changed from relatively dispersed to more pronounced local concentration. Consistent with the numerical results, peak contact pressure increased continuously from 2.55 MPa to 3.14 MPa, indicating that along with spatial migration, the degree of local contact-load concentration also continued to intensify.

Overall, the spatial migration pattern of peak contact pressure on the distal tibial articular surface can be summarized as follows: as the external-rotation moment increased, the hotspot of peak contact pressure continuously migrated from the anterolateral to the anteromedial region and formed a more localized high-pressure concentration zone in the high-moment phase. Combined with the numerical changes, peak contact pressure increased continuously overall, but the growth rate was relatively slow within 0–2.4 N·m and became faster beyond 2.6 N·m, suggesting a further increase in the risk of local contact-load concentration under higher external-rotation moments.

## 5 Discussion

### 5.1 Principal findings

Using a patient-specific ankle finite element model under a closed-chain single-leg stance loading condition, this study analyzed the local mechanical response of the distal tibial articular surface by applying graded talar external-rotation moments as controlled surrogate perturbations. The results showed that different mechanical indicators responded differently to increasing external-rotation moments. Specifically, peak surface stress on the distal tibial articular surface exhibited a clear nonlinear pattern, decreasing progressively within 0–2.2 N·m, reaching its minimum at 2.2 N·m, and then increasing again as the moment continued to rise. In contrast, peak contact pressure showed no decrease but instead increased continuously with higher external-rotation moments, and the increase became more pronounced at high moment levels. At the same time, the change in the relative rotation angle of the talus with respect to the tibia about the *Z* axis increased continuously and monotonically with increasing moment, indicating that the current loading approach generated stable and measurable axial rotational responses in the model.

In terms of spatial distribution, the two indicators also showed different characteristics. The high-stress region of peak surface stress migrated overall from the anterolateral to the anteromedial region, with the peak hotspot shifting from lateral to medial between 2.6 and 2.7 N·m. In contrast, the hotspot migration of peak contact pressure was more clearly defined and showed a progressive inward concentration from lateral to medial. These findings suggest that, under the present closed-chain compressive condition, talar external rotation not only alters local articular stress levels but also reshapes the position and concentration of the principal tibiotalar contact region.

### 5.2 Differential responses of local surface stress and contact loading on the distal tibial articular surface under external-rotation moments

One of the most notable findings of this study is that peak surface stress and peak contact pressure did not respond synchronously to graded external-rotation moments. Intuitively, if articular loading were “relieved,” one might expect both peak stress and peak contact pressure to decrease simultaneously; however, this was not the case in the present model. Peak surface stress decreased within the range of 0–2.2 N·m, whereas peak contact pressure increased gradually over the same interval. This indicates that under the present modeling conditions, moderate talar external rotation was more likely to produce a redistribution of the local surface stress state rather than “contact unloading” in the strict sense of contact-pressure reduction.

From a mechanical perspective, peak contact pressure more directly reflects the degree of local concentration of normal articular contact load, whereas peak surface stress reflects the combined stress state at local material points and is jointly influenced by local geometry, changes in contact extent, and multiaxial stress redistribution. Therefore, when the talus underwent moderate external rotation, structural stress in some regions along the anterior articular margin may have been redistributed and thus peak surface stress temporarily decreased; however, the local normal contact load did not decrease accordingly and instead rose gradually. As the moment further increased beyond 2.6–3.0 N·m, the increase in peak contact pressure became more obvious, while peak surface stress also rose again, indicating that local articular stress redistribution had gradually transitioned into a new pattern of local load concentration.

This interpretation is supported by the spatial migration results. With continued talar external rotation, the high-pressure region of peak contact pressure continuously concentrated from the anterolateral toward the anteromedial side, indicating that the principal load-bearing contact region was being progressively repositioned and concentrated in a more medial local area. In contrast, the high-stress region of peak surface stress also showed an overall medial shift, but the cross-regional change in its maximum location occurred in the interval between 2.6 and 2.7 N·m, when the peak hotspot shifted from the lateral to the medial side. This suggests that, with increasing external-rotation moment, the main concentration point of local structural stress on the articular surface underwent a lateral-to-medial transition.

Taken together, the numerical results and spatial migration patterns suggest that, under the present loading scenario, peak surface stress on the distal tibial articular surface exhibited a relative low-value interval, which may be regarded as a “relative minimum-stress window” for the articular surface. By contrast, although peak contact pressure increased overall with increasing moment, its growth was relatively slow in the low-to-moderate moment range, indicating that the degree of contact-load concentration had not yet increased markedly during that phase and became more pronounced only at higher moment levels.

### 5.3 Implications for clinical practice and future research

In the context of clinical application, the value of the present study lies more in providing mechanistic insight than in directly prescribing intervention parameters. For ankle and foot disorders such as OLT, CAI, and AAFD, which share talar malalignment or abnormal talar biomechanics as common features, the results suggest that it is insufficient to judge an intervention simply by whether it reduces the overall joint load. Rather, it is necessary to distinguish between two different mechanical indicators: local structural stress state and local contact-pressure concentration. Certain intervention parameters may reduce peak surface stress within a certain range while not reducing peak contact pressure, and may even gradually increase it. This means that the goal of clinical intervention should not be simplistically summarized as “the more external rotation the better” or “the more wedging the better,” but should instead be understood as a multi-indicator optimization problem.

For OLT, both articular cartilage and subchondral bone are highly sensitive to local contact-pressure concentration. Therefore, if an intervention improves certain surface stress states while peak contact pressure continues to rise, its protective significance should be interpreted cautiously. For CAI, the present findings suggest that talar axial rotation and the associated repositioning of the contact region may be important factors influencing the local loading environment of the ankle, rather than merely superficial manifestations of postural abnormality or plantar pressure change. For AAFD and other disorders associated with abnormal talar internal/external rotation, the present study provides a way of understanding external orthotic interventions from the perspective of the local tibiotalar contact environment: the significance of parameter adjustment lies not only in altering lower-limb alignment or gait characteristics, but also in reshaping the location of the articular load center and the pattern of load concentration.

It should be emphasized that the relatively low-stress interval of 2.0–2.4 N·m identified in this study only indicates that peak surface stress was relatively low under the present model and static closed-chain loading scenario; it cannot be directly extrapolated as the optimal clinical correction range. Its real significance lies in showing that graded talar external-rotation perturbation has a nonlinear relationship with local articular mechanical indicators and that different indicators may exhibit dissociated changes. Accordingly, future higher-fidelity studies should further test whether the phenomenon of “decreasing structural stress but increasing contact pressure” persists under loading conditions that are closer to physiological reality.

### 5.4 Strengths and limitations

This study employed a patient-specific three-dimensional ankle finite element model and compared graded talar external-rotation moments under unified boundary conditions, allowing a systematic observation of dose-response relationships, which are difficult to achieve using traditional experiments. This study also reported not only numerical changes but also the spatial migration characteristics of peak surface stress and peak contact pressure based on contour maps, thereby avoiding interpretation of complex joint contact behavior solely on the basis of single peak values. Finally, the study additionally extracted the relative rotation angle of the talus with respect to the tibia, establishing a supplementary kinematic link between moment loading and changes in joint posture.

At the same time, several limitations should be acknowledged. First, the present model was based on only one healthy subject and therefore cannot represent the talar morphology, ankle mortise geometry, or soft-tissue conditions that may exist in different pathological populations, limiting the generalizability of the results. Second, the study did not explicitly model orthotic geometry, plantar contact, or actual external intervention structures; instead, graded talar external-rotation moments were used as surrogate variables. Therefore, the results are better suited to answering how talar external rotation affects the local mechanical environment of the tibiotalar joint than to being directly translated into clinical quantities such as “how many millimeters of wedging” or “what support angle should be prescribed.” Third, the study was conducted under a static closed-chain single-leg stance condition and did not include dynamic tasks such as gait, heel-rise, or stair descent, nor did it incorporate muscle forces or other physiological loads. As a result, the relative low-stress interval and hotspot migration patterns identified here may shift under dynamic conditions. Fourth, articular cartilage was modeled as a simplified continuous body generated from bony articular surfaces and treated as an isotropic linear elastic material with low-friction frictional contact (coefficient of friction = 0.0025), while ligaments were represented by linear spring elements without explicit incorporation of nonlinear constitutive behavior, prestress, or viscoelasticity. Because the primary outcomes of the present study were articular-surface stress- and contact-pressure–related metrics, this simplification is not trivial. In particular, the use of a linear elastic cartilage model means that the specific absolute values of the reported stress/pressure metrics are unlikely to represent the true tissue-level mechanical behavior of native cartilage with high fidelity. Therefore, the numerical magnitudes reported in this study should be interpreted cautiously. By contrast, under the present unified modeling framework, the comparative patterns observed across graded loading conditions-including the relative increases/decreases in peak surface stress and peak contact pressure, as well as their spatial migration characteristics-are likely to be more robust and more informative than the absolute values themselves.

Future studies may extend the present framework in two directions. First, the model may be advanced from a “surrogate-variable model” toward a “clinical-parameter model” by incorporating medial wedge insole geometry, plantar contact, and more realistic hindfoot coupled motion to improve correspondence between the model and clinical intervention. Second, dynamic tasks, muscle loading, and larger numbers of subjects should be introduced to verify whether the mechanical trends observed here remain consistent under more complex and diverse physiological conditions.

## 6 Conclusion

Under a closed-chain single-leg stance compressive condition, talar external rotation can induce a marked redistribution of the local mechanical environment on the distal tibial articular surface. peak surface stress exhibits a relatively low-stress interval, whereas peak contact pressure increases continuously overall, with only a relatively slow rate of increase in the low-to-moderate moment range. The high-value regions of both metrics migrate from the anterolateral to the anteromedial region, suggesting that talar external rotation simultaneously influences the local structural stress state and the concentration pattern of articular contact loading.

## Data Availability

The data supporting the findings of this study are available from the corresponding author upon reasonable request. The raw CT imaging data are not publicly available due to ethical and privacy restrictions.
